# High-Fat Diet Alters the Orosensory Sensitivity to Fatty Acids in Obesity-Resistant but not Obesity-Prone Rats

**DOI:** 10.4172/1747-0862.1000168

**Published:** 2015-03-11

**Authors:** David W Pittman, Dane R Hansen, Timothy A Gilbertson

**Affiliations:** 1 Department of Psychology, Wofford College, USA; 2 Department of Biology, Utah State University, USA

**Keywords:** Dietary fat, Fatty acids, Taste sensitivity, Obesity phenotypes, Rat models

## Abstract

Gene-environment interactions play a role in the development of obesity but specific effects of diet on the orosensory detection of fatty acids have yet to be clarified. The objective of this study is to characterize the effect of prolonged (5-week) exposure to a high-fat (60%) diet on the behavioral sensitivity to the fatty acid linoleate following a conditioned taste aversion in obesity-prone and obesity-resistant rats. Exposure to the high-fat diet significantly enhanced the sensitivity of obesity-resistant (S5B/Pl) rats to linoleate while producing no effect on the fatty acid sensitivity for obesity-prone rats. Specifically, high-fat diet fed S5B/Pl rats showed stronger initial avoidance of linoleate and slower extinction rates than their normal diet cohorts. Our study suggests that prolonged dietary fat consumption may alter the behavioral sensitivity to fatty acids particularly in obesity-resistant animals.

## Introduction

The prevalence of obesity continues to grow along with health risks associated with this chronic condition such as cardiovascular disease, hypertension, diabetes, cancer, and other nutrition-related disorders [1,2]. The principal contributors to the formation of obesity have been identified as physical inactivity [3,4], diet [5–8], and genetic traits [9–12]. It is also clear that interactions between environment and genes are important in the etiology of obesity and that the consumption of high fat food represents a major environmental factor that may influence physiological mechanisms in a manner that increases susceptibility to obesity [11,13–16].

Mounting evidence has shown that rats [17] and humans [18,19] can detect the presence of fatty acids, the chemical components of dietary fat, in the oral cavity most likely through taste-mediated cues. Linoleic acid, or its aqueous form sodium linoleate, appears to be the most salient of the fatty acids commonly found in dietary fat [20–25]. Recent reports indicate that humans may be either hyposensitive or hypersensitive to the behavioral detections of dietary fat in the oral cavity and that obese humans may have a reduced fat taste sensitivity [26,27]. Furthermore using analogous animal models, we have shown that obesity-prone and obesity-resistant strains of rat exhibit differential sensitivity to fatty acid detection following a conditioned taste aversion. Obesity-prone (Osborne-Mendel, OM) rats appear more behaviorally sensitive to fatty acids showing stronger conditioned taste aversions with slower extinction rates for linoleate than obesity-resistant (S5B/Pl) rats [22] and OM rats show stronger behavioral preferences for linoleic acid than S5B/Pl rats [28]. Fatty acids are transduced by multiple receptors on the tongue such as CD36, a host of G protein-coupled receptor (GPR)-mediated pathways (GPR40, GPR41, GPR43, GPR84, and GPR120) and delayed rectifying potassium (DRK) channels [29]. While there does not appear to be differential expression of CD36, GPR40, GPR120 between the OM and S5B/Pl strains [28], there is differential expression of fatty acid-sensitive and fatty acid-insensitive DRK channels between the two strains such that S5B/Pl rats have a greater ratio of fatty acid-sensitive to insensitive DRK channels (5.2:1) than OM rats (1.7:1) [30]. This differential expression of fatty acid-sensitive DRK channels is predicted to lead to greater taste receptor cell activation by fatty acids in the S5B/Pl strain compared to the OM strain [30]. The consumption of dietary fat appears to modulate the sensitivity to fatty acid detection with prolonged exposure to a high-fat diet reducing the expression of fatty acid-sensitive DRK channels in the S5B/Pl rat (5.2:1 to 0.65:1) along with a corresponding reduction in the electrophysiological responsiveness of S5B/Pl taste receptor cells to fatty acid stimulation [31].

Thus, it appears that prolonged exposure to a high-fat diet results in S5B/Pl rats that now exhibit ratios of fatty acid-sensitive:fatty acid-insensitive DRK channels similar to OM rats on a regular diet. Given that OM rats on a normal diet showed greater behavioral sensitivity to fatty acids following a conditioned taste aversion than S5B/Pl rats, we predicted that S5B/Pl rats maintained on a high-fat diet would show increases in their behavioral sensitivity to fatty acids in a similar conditioned taste aversion test. This study examines the influence of a high-fat diet compared to normal rodent chow on the sensitivity of OM and S5B/Pl rats to the fatty acid linoleate following a conditioned taste aversion.

## Methods and Procedures

Male OM and S5B/Pl rats (161–168 days old at testing) were maintained on either normal rodent chow (Teklad 8604, 4% fat) or a high-fat chow (Research Diets D12492, 60% fat) for a 5-week period prior to and during testing. Each group was further subdivided into experimental groups receiving injections of either LiCl (OM-Reg n=10, OM-Fat n=5, S5B/Pl-Reg n=10, S5B/Pl-Fat n=5) to induce a conditioned taste aversion or saline (OM-Reg n=10, OM-Fat n=5, S5B/Pl-Reg n=9, S5B/Pl-Fat n=5) as a control on the conditioning days. All rats were maintained on 23-hr water restriction schedules beginning 3 days prior to conditioning and testing. All animal procedures were conducted in accordance with the NIH guidelines and were approved by the Institutional Animal Care and Use Committee of Wofford College.

For three consecutive conditioning days, rats received 10 min access to the conditioned stimulus (CS), 100 μM linoleate (Sigma-Aldrich, St. Louis, MO) in deionized water, 20 minutes prior to receiving their designated unconditioned stimulus of either 150 mM LiCl or saline at a dosage of 10 ml/kg. Approximately 20 minutes following the injections, all rats receiving LiCl showed signs of gastric malaise (lethargic symptoms: no observed feeding, grooming or rearing behavior) while all saline-injected animals showed normal levels of activity. Consumption of the linoleate during conditioning was measured by bottle weight. All rats received supplemental access to water in their home cage for 45 min approximately 6 hours after the conditioning trial.

Testing began the day after the third conditioning trial. Single daily test sessions for three consecutive days assessed the formation and extinction of a taste aversion to linoleate in the MS-160 gustometer as previously described [21,32]. Test sessions consisted of 4 blocks of stimulus trials 15 seconds in duration with 15 second interstimulus intervals. Linoleate at 5, 20, 50, and 100 μM concentrations and a water stimulus were presented in randomized order once in each of the 4 blocks of stimulus trials. The cumulative licks per trial for each stimulus were averaged across the 4 stimulus blocks. Lick ratios (licks for CS stimulus/average licks for water) were calculated in order to normalize the linoleate licking responses to the water licking response for each rat. The latency until the first lick in each trial was measured as an indicator of whether olfactory cues were potentially used by rats to avoid consumption of the stimuli. A mixed factorial ANOVA with post-hoc pairwise comparisons (Bonferroni) were used to identify statistically significant (p < 0.05) main effects and interactions between independent variables.

## Results

A significant main effect of strain [F(1,55)=20.9, p<0.001] and diet [F(1,55)=128.8, p<0.001] was observed for body weight with no significant interaction between the two variables. The body weight of the OM rats (312.7 ± 10.1 g) was greater than the S5B/Pl rats (241.8 ± 10.4 g) when fed regular chow and both the OM rats (440.1 ± 14.3 g) and the S5B/Pl rats (396.9 ± 14.1 g) fed the high-fat diet gained more weight than their regular chow cohorts. To determine whether or not exposure to a high-fat diet for 5-weeks affected the orosensory sensitivity to fatty acids of rats, we examined their avoidance of linoleic acid during brief-access gustatory tests following three conditioning days. Regardless of dietary condition, similar and significant reductions in linoleate (CS) consumption from conditioning day 1 to day 2 were observed for the OM (regular chow: t(9) = 11.8, p < 0.01; high-fat chow: t(4) = 15.2, p < 0.01) and S5B/Pl (regular chow: t(9) = 9.9, p < 0.01; high-fat chow: t(4) = 10.9, p < 0.01) rats receiving LiCl injections as the unconditioned stimulus. There was no difference in linoleate consumption between conditioning days 2 and 3 for any of the LiCl-injected groups indicating no differences between strains in the ability to form a conditioned taste aversion following a single pairing. There was no difference in linoleate consumption across the 3 conditioning days for the saline-injected groups. The latency until the first lick did not significantly differ between the experimental groups or across concentration or test day indicating that rats did not use olfactory cues to avoid approaching and licking any specific stimuli.

**Figure 1:**
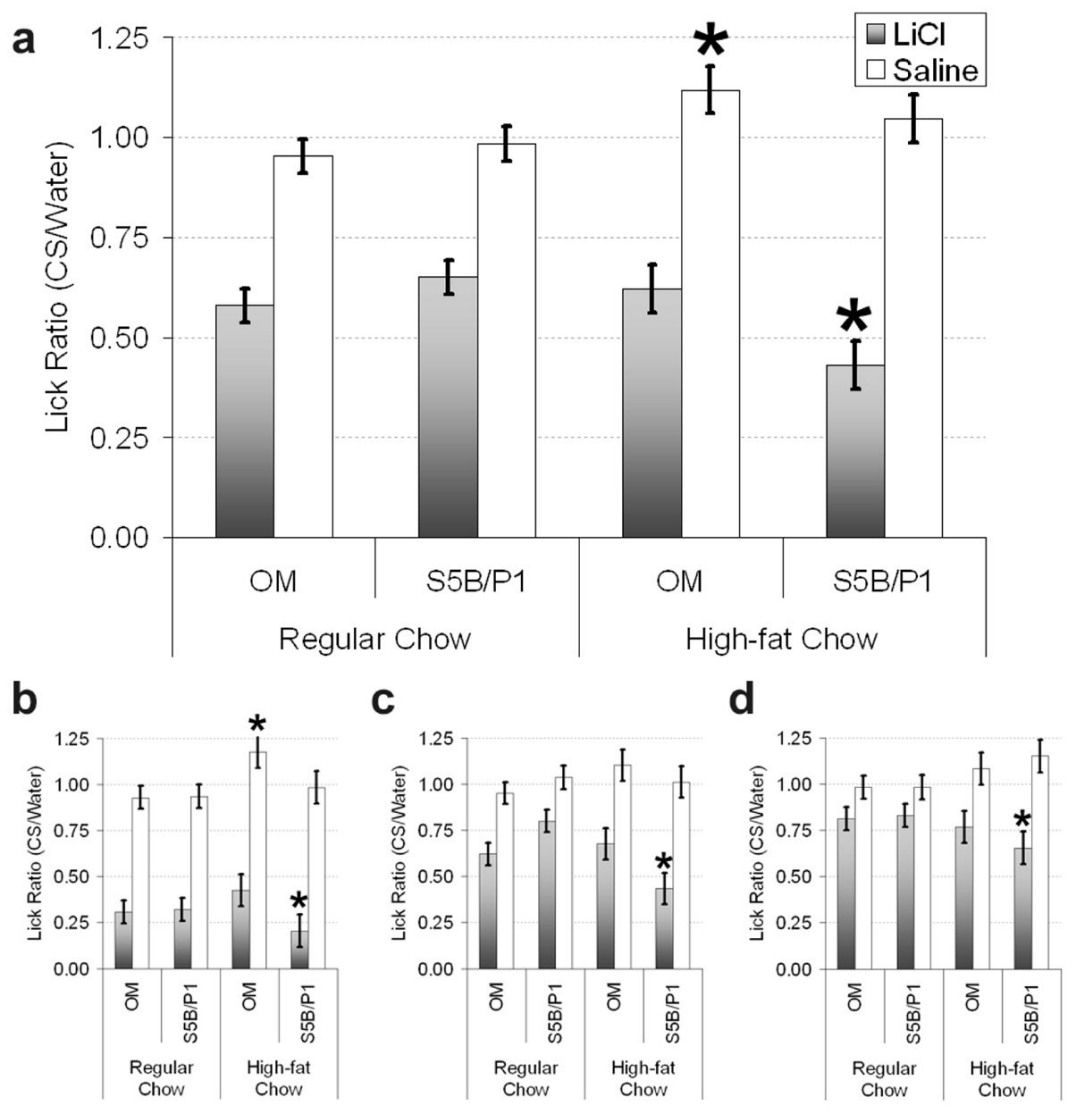
Consumption of linoleate (CS) following a conditioned taste aversion in obesity-prone (OM) and obesity-resistant (S5B/Pl) rats maintained on regular chow or high-fat chow for 5-weeks prior to testing. (a) Avoidance of linoleate collapsed across testing days and concentration. Avoidance of linoleate collapsed across concentration for test day 1 (b), 2 (c), and 3 (d). Significant (p < 0.01) effects of the diet condition within strain and injection conditions are indicated by asterisks. LiCl, conditioned taste aversion; Saline, control group.

As shown in Figure 1a, collapsed across linoleate concentration and test day, there was a significant main effect of injection [F (1,51) = 156.7, p < 0.001] and furthermore there was a significant 3-way interaction between injection, strain of rat, and diet [F(1,51) = 6.3, p < 0.01]. This indicates that not only did the LiCl-injected groups avoid linoleate but that the dietary condition produced differential effects for the strains within the injection conditions. Post-hoc pairwise comparisons revealed the source of this interaction to be a significant increase in avoidance for the S5B/Pl LiCl-injected rats maintained on the high-fat diet compared to the S5B/Pl LiCl-injected rats on normal chow (p < 0.01). There was also a significant increase in the consumption of linoleate by the OM saline-injected rats maintained on the high-fat diet compared to the OM saline-injected rats on the normal chow (p < 0.01). The increased avoidance of linoleate by the S5B/Pl rats on the high-fat diet is accounted for by stronger avoidance on day 1 of testing (Figure 1b) and greater resistance to extinction on testing days 2 and 3 (Figure 1c and d). The significant difference in extinction is confirmed by a 5-way interaction [F (6,306) = 2.4, p < 0.05] between the variables: test day, concentration of linoleate, diet, strain, and injection conditions.

## Discussion

The ability of a prolonged high-fat diet to increase fatty acid sensitivity in S5B/Pl rats following a conditioned taste aversion was predicted based on previously reported effects of high-fat diet exposure on the expression of DRK channels in S5B/Pl taste receptor cells [31]. We had also previously reported that OM rats maintained on a normal diet showed stronger taste aversions and slower extinction times than S5B/Pl rats on a normal diet. This behavioral difference corresponded with a reduction in the ratio of fatty acid-sensitive to insensitive DRK channels for OM rats (1.7:1) compared to S5B/Pl (5.2:1). Whereas, prolonged exposure to a high-fat diet altered the S5B/Pl ratio of fatty acid-sensitive to insensitive DRK channels (0.65:1) such that this strain now expressed a lower ratio of fatty acid sensitive DRK channels than the OM rats on a normal diet. This change in DRK channel expression corresponds with behavioral results showing that when S5B/Pl rats are maintained on a high-fat diet their sensitivity to avoiding linoleate increases in a manner surpassing the normal-diet OM rats. Specifically, the initial aversion was stronger and more resistant to extinction on the second day of testing for the S5B/Pl rats on the high-fat diet compared to S5B/Pl and OM rats fed a normal diet.

Interestingly, there was no observed effect of the high-fat diet on the avoidance of linoleate for the OM rats; however, there was an increase in the innate preference for linoleate in the high-fat diet OM rats receiving saline injections. This control group of OM rats fed the high-fat diet licked on average 12% more to the linoleate stimuli than water during testing, whereas their normal-diet cohorts licked the linoleate stimuli 5% less than water. This suggests that exposure to a high-fat diet may influence the preference for fatty acids without affecting the physiological mechanisms underlying the detection sensitivity for fatty acids in the OM strain.

Our findings are the initial exploration into the ability of dietary fat content to manipulate fatty acid orosensory detection thresholds with the potential to alter ingestive responses to fatty acids. No observed effects on the latency to lick and our brief-access testing methods minimize the potential confounds of alterations in both olfactory and post-ingestive signal pathways in response to prolonged high-fat diet consumption. It appears that exposure to a high-fat diet alters the orosensory behavioral sensitivity of obesity-resistant rats to become more sensitive in detecting fatty acids than obesity-prone rats. While corresponding changes in DRK expression due to high-fat diet may play a role in the shift of behavioral sensitivity, it is likely that yet to be identified alterations of additional physiological mechanisms related to fatty acid detection such as CD36 and G protein-coupled receptors may also contribute to changes in the behavioral responsiveness to fatty acids following prolonged exposure to a high-fat diet.
